# Central Projections of Antennal Sensory Neurons in the Aphid *Myzus persicae*

**DOI:** 10.3390/insects17030249

**Published:** 2026-02-27

**Authors:** Baiwei Ma, Jing Li, Feiyue Ding, Xi Chu, Xiaoyan Zhu, Shuai Liu, Guirong Wang, Qi Chen, Bingzhong Ren

**Affiliations:** 1 Jilin Provincial Key Laboratory of Animal Resource Conservation and Utilization, School of Life Sciences, Key Laboratory of Vegetation Ecology, Ministry of Education, Northeast Normal University, Changchun 130024, China; mabaiweilmr@163.com (B.M.); fyding657@nenu.edu.cn (F.D.); zhuxy575@nenu.edu.cn (X.Z.); 2 Shenzhen Branch, Guangdong Laboratory for Lingnan Modern Agriculture, Genome Analysis Laboratory of the Ministry of Agriculture, Agricultural Genomics Institute at Shenzhen, Chinese Academy of Agricultural Sciences, Shenzhen 518120, China; 3 State Key Laboratory for Biology of Plant Diseases and Insect Pests, Institute of Plant Protection, Chinese Academy of Agricultural Sciences, Beijing 100193, China; 4 Research Institute of Forest Protection, Jilin Provincial Academy of Forestry Sciences, Changchun 130033, China; lij9030@163.com; 5 Jilin Province Main Forest Diseases and Insect Pests Monitoring, Epidemic Prevention Technology Innovation Center, Changchun 130033, China; 6 Chemosensory Lab, Department of Psychology, Norwegian University of Science and Technology, 7491 Trondheim, Norway; xi.chu@ntnu.no; 7 Department of Plant Protection, Jilin Agricultural University, Changchun 130118, China; liushuai0903@126.com

**Keywords:** antenna, antennal lobe, brain, ventral nerve cord, olfaction, *Myzus persicae*

## Abstract

The aphid *Myzus persicae* is an important agricultural pest relying on olfactory cues to find food and escape from predators. The considerable difficulty of anatomical manipulation and other related technical challenges may underlie the absence of studies systematically mapping the central projections of antennal sensory neurons in its central nervous system. We used mass stain, immunohistochemistry, laser scanning confocal microscope and three-dimensional reconstruction techniques to trace the central projections of sensory neurons from the antennae. The ipsilateral antennal nerve targets mainly the antennal lobe, the antennal mechanosensory and motor center, and most neuromeres of the ventral nerve cord. There are also several axons that project to other regions, like the contralateral antennal lobe, the contralateral antennal nerve, the ipsilateral calyx and some region of the protocerebrum. The numerous neuropils innervated by axons from the antenna indicate the multiple roles that this sensory organ serves in insect behavior, and the results which provide information about the basic anatomical arrangement of the olfactory nervous system in aphid *M. persicae* may provide some basic knowledge for the further investigations of the aphid.

## Simple Summary

This study investigated the central projections of antennal neurons in the aphid *Myzus persicae*. Our results show that these neurons primarily project to the ipsilateral antennal lobe and antennal mechanosensory and motor center, with some fibers projecting to the ventral nerve cord, the calyx, the protocerebrum, the contralateral antennal lobe and the contralateral antennal nerve. The numerous neuropils innervated by antennal neural axons suggest the multiple roles of this sensory organ in aphid behavior. These findings provide information about the anatomical arrangement of the olfactory nervous system in *M. persicae* and serve as a basis for future research on aphid sensory processing.

## 1. Introduction

Aphids pose a significant threat to agricultural production, leading to direct damage to crop and potentially spreading plant diseases [1]. Like other insects, olfaction plays a crucial role in the sensory perception of aphids [2]. For instance, they can swiftly relocate from their original positions upon detecting alarm pheromones, a mechanism designed to evade predation by predators [3,4,5]. It is worth noting that olfaction in insects is pivotal not only in predator avoidance, but also in various other behaviors, such as locating food sources, finding mates, and identifying suitable sites for egg laying [6,7]. Antennae serve as the primary olfactory organ in most insects, including aphids, housing numerous sensilla and many of which contain olfactory sensory neurons (OSNs) that predominantly project to the antennal lobe (AL) [8,9]. OSNs play a crucial role in detecting odorant molecules in the periphery and transmitting signals through axons to the AL. Within the AL, the processing of odorant information involves local neurons (LNs) and projection neurons (PNs), along with glomeruli, which are recognized as functional units in olfactory information processing. Following this processing, signals are conveyed to the higher brain centers through PN axons, ultimately resulting in the generation of motor commands [8,9,10].

Knowing the anatomical structure of the olfactory system is a prerequisite for understanding how odor information is encoded and decoded, and this knowledge will help to predict the biological significance to a given odor. Functional research using optical imaging methods on fruit flies, moths, bees and others has shown that odors are encoded as specific spatio-temporal glomerular activation patterns, often with several glomeruli responding to a given odor component [11,12,13,14,15,16]. Thus, the establishment of the AL three-dimensional atlas in various model species has been used as a basis to interpret data from functional studies [17,18,19,20,21,22,23,24,25,26,27,28,29,30,31].

Probably due to aphids’ small body size, soft texture, fragile neural tissue, and the close proximity of extensive head musculature to the brain, very few studies have yet illustrated their antennal lobe structure [32,33,34]. In this study, we investigated the central projection of the antennal nerve in the aphid *M. persicae*. Utilizing a combination of synaptic antibody immunostaining and mass staining of antennal nerve together with computerized 3D reconstruction, we generated a moderately detailed three-dimensional atlas that includes the olfactory system and their innervating regions.

## 2. Materials and Methods

### 2.1. Insects

Adult *M. persicae* were collected from the peach tree *Prunus davidiana* during local fieldwork. The specific location for the collection was within the park of the Institute of Food Science and Technology, Chinese Academy of Agricultural Sciences, located in Beijing, China, spanning the months of April to June.

### 2.2. Mass Stain of Antennal Sensory Neurons

The aphid was immobilized within a small dental wax hole, providing a suitable containment for its size. The aphid head capsule was covered with dental wax, and a small wax wall was formed around one of the antennae. To ensure a seal between the wax wall and the aphid head capsule part around the antenna, the wax wall was gently melted by a modified low-temperature cautery. The antenna was cut off from its base, and a small amount of water was added into the tiny pool. Crystals of fluorescent dye Micro-Ruby (TMR; tetramethylrhodamine dextran with biotin, Micro-Ruby, Molecular Probes (Eugene, OR, USA); Invitrogen, Eugene, OR, USA) were subsequently introduced into the water pool and the pool was sealed by Vaseline. All post-operative aphids were kept in a dark box with high-moisture environment at 4 °C for about 12 h or overnight. Dissections of the central nervous system were subsequently performed in Ringer’s saline (in mM, 150 NaCl, 3 CaCl_2_, 3 KCl, 25 sucrose, and 10 TES buffer, pH 6.9). The samples were then fixed in fresh 4% paraformaldehyde solution (4% PFA) in phosphate-buffered saline (PBS; in mM, 137 NaCl, 2.7 KCl, 10 Na_2_HPO_4_, 1.8 KH_2_PO_4_, pH 7.4) for 2 h or overnight at a room temperature or 4 °C, respectively.

### 2.3. Immunohistochemistry

The anti-synapsin antibody (SYNORF1; catalog No 3C11; RRID: AB_2313867; Developmental Studies Hybridoma Bank [DSHB], University of Iowa, Iowa City, IA, USA) was used to label the neuropil structures. Briefly, the samples of central nervous system underwent a series of washes in PBS with 1% Triton X-100 (PBST) for 4 × 15 min. Thereafter, the samples were pre-incubated in 5% normal goat serum (NGS; Sigma, St. Louis, MO, USA) in PBST at room temperature for 3 h before immersing in 1% primary antibody SYNORF1 in PBST at 4 °C for 3 days. Then, the samples were incubated in 0.4% secondary antibody, Cy2-conjugated anti-mouse (Cy2; Invitrogen, Eugene, OR, USA) in PBST at 4 °C for 2 days after rinsing for 6 × 20 min in PBST. Finally, the samples were rinsed again for 6 × 20 min in PBST, dehydrated with an ascending ethanol series (50%, 70%, 90%, 95%, 100% × 2, 10 min each), cleared in xylene and mounted in ZEISS Immersion Oil (n_e_ = 1.518).

### 2.4. Laser Scanning Confocal Microscopy

Labeled samples were visualized by using a laser scanning confocal microscope (LSM 980 META Zeiss, Jena, Germany) equipped with Zeiss Plan-Apochromat 63×/1.40 oil objective and Plan-Apochromat 20×/0.8 objective. A HeNe1 laser 546 nm line was used to excite the Micro-Ruby, and a 488 nm line of argon laser was used to excite the Cy2. The images were obtained with a resolution of 512 × 512 pixels in the *xy* plane and an interslice distance of 1–2 µm.

### 2.5. Three-Dimensional Reconstructions

To make a 3D atlas of the central projection of the antennal nerve in the central nervous system, we used Amira 5.3 (Visage Imaging, Fürth, Germany) for the reconstruction of confocal image stacks. The central nervous system, neuromeres, AL and AMMC (antennal mechanosensory and motor center) were labeled by using the segmentation editor, including the Brush and Interpolate tools. The labeling of the central projecting antennal nerve was achieved through the application of the SkeletonTree editor in Amira 5.3.

## 3. Results

### 3.1. Overview of the Antennal-Axon Terminals in the Central Nervous System

**Figure 1:**
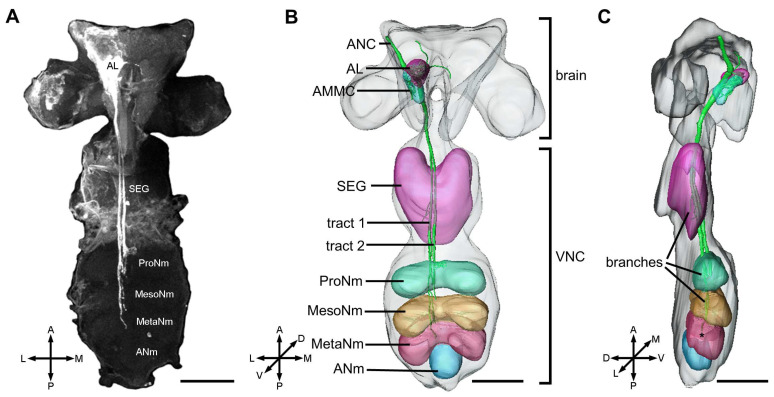
Gross projection pathway of the antennal neurons in the central nervous system of *M. persicae*. (**A**): Confocal image of antennal axons terminating in the brain and ventral nerve cord. (**B**,**C**): Three-dimensional reconstruction showing the projection pathway from the antenna into the central nervous system (frontal and lateral view, respectively). ANC, antennal nerve cord; AL, antennal lobe; AMMC, antennal mechanosensory and motor center; SEG, subesophageal ganglion; ProNm, prothoracic neuromere; MesoNm, mesothoracic neuromere; MetaNm, metathoracic neuromere; ANm, abdominal neuromere; VNC, ventral nerve cord; A, anterior; D, dorsal; L, lateral; M, medial; P, posterior; V, ventral. Scale bars = 50 μm.

Probably due to aphids’ small body size, soft texture, fragile neural tissue, and close proximity of extensive head musculature to the brain, only six central nervous systems were successfully dissected from 30 aphids processed for staining; four of these were successfully labeled with the neural tracer (Micro-Ruby) and used for subsequent analyses in this study. Our observation revealed that the antennal neuron fibers extending through the antennal nerve cord project predominantly to the ipsilateral AL and AMMC, as well as the subesophageal ganglion (terminology was referenced from Kollmann et al. [33]), prothoracic, mesothoracic, and metathoracic neuromeres (Figure 1).

### 3.2. Central Projection Pattern of Antennal Sensory Neurons Within the Brain

**Figure 2:**
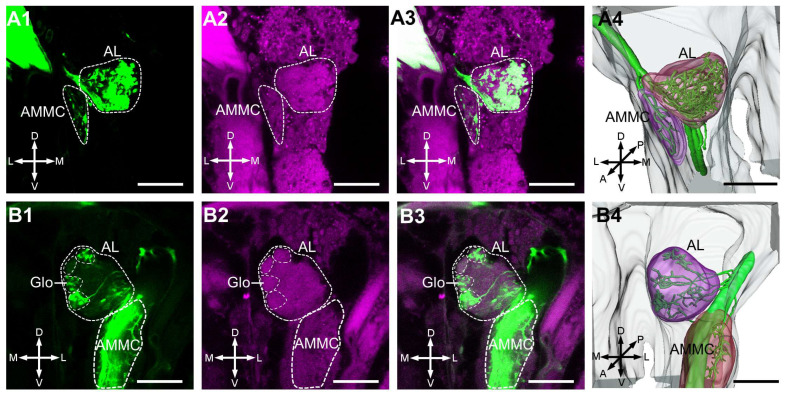
Central projections of antennal neurons in the AL and AMMC. Green and magenta denote neural fibers and synapsin protein-immunostained tissues, respectively, in (**A1**–**A3**,**B1**–**B3**). (**A1**–**A3**): Confocal images of antennal neurons targeting the AL and AMMC (dotted white line). (**A4**): Three-dimensional reconstruction of the antennal neurons to the AL and AMMC. (**B1**–**B4**): In one sample, glomeruli can be viewed by the outline of axon terminals, but the outline based on anti-synapsin staining is fuzzy. AL, antennal lobe; AMMC, antennal mechanosensory and motor center; Glo, glomeruli; A, anterior; D, dorsal; L, lateral; M, medial; P, posterior; V, ventral. Scale bars = 20 μm.

Our data illustrated that the antennal axons mainly project to the AL and innervate most glomeruli. However, in all collected samples, there is an absence of anti-synapsin labeling for the glomerular structure in the aphid AL (Figure 2(A1–A4)). Although we clearly observed antennal axons that outline the glomerular structures in one sample, the anti-synapsin labeling of glomerular boundaries remains fuzzy (Figure 2(B1–B4)). In the AMMC region, located posterior to the AL, we observed branches from the antennal nerve providing innervation (Figure 2(B1–B4)).

### 3.3. Projection Pattern of Antennal Neurons in the Subesophageal Ganglion

**Figure 3:**
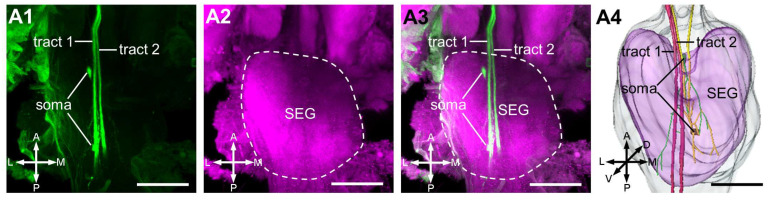
Central projections of the antennal neurons in the SEG. (**A1**–**A4**): Two tracts (tract 1 and tract 2) project in the SEG and two somas are in this region. SEG, subesophageal ganglion; A, anterior; D, dorsal; L, lateral; M, medial; P, posterior; V, ventral. Scale bars = 50 μm.

Two parallel fiber bundles were observed projecting to the subesophageal ganglion (Figure 3), with only a minimal number of branches extending from these bundles to the neuromere. 

### 3.4. Projection Pattern of Antennal Neurons in the Ventral Nerve Cord

**Figure 4:**
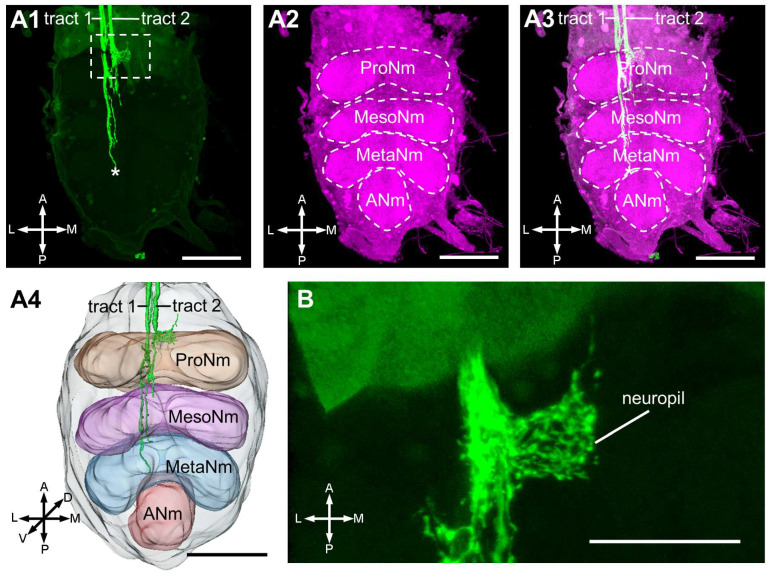
Central projections of the antennal neurons in the thoracic ganglia. (**A1**): The two antennal nerve axon tracts project in the thoracic ganglia and one tract terminals (indicates by the asterisk) in the MetaNm. (**A2**): The thoracic ganglia have four neuromeres, namely ProNm, MesoNm, MetaNm, and ANm. (**A3**): The merge of central projections of antennal neurons and anti-synapsin immunostaining result of the thoracic ganglia. (**A4**): Three-dimensional reconstruction of the antennal neurons in the thoracic ganglia. (**B**): There is a neuropil structure innervated by tract 2 in the ProNm. ProNm, prothoracic neuromere; MesoNm, mesothoracic neuromere; MetaNm, metathoracic neuromere; ANm, abdominal neuromere; A, anterior; D, dorsal; L, lateral; M, medial; P, posterior; V, ventral. Scale bars = 50 μm in (**A1**–**A4**). Scale bar = 20 μm in (**B**).

There are two parallel fiber bundles (Tract 1 and 2) targeting the ventral nerve cord through all neuromeres except the abdominal neuromere. Tract 1 innervates through the prothoracic, mesothoracic, metathoracic neuromeres, with a few fibers projecting to the three neuromeres (Figure 4(A1–A4)). Conversely, almost all fibers of Tract 2 project specifically to the prothoracic neuromere, forming a distinct neuropil structure (Figure 4B). The trajectory of Tract 2 halts in the mesothoracic neuromere.

### 3.5. Secondary Projection Pattern of Antennal Neurons in the Ventral Nerve Cord

**Figure 5:**
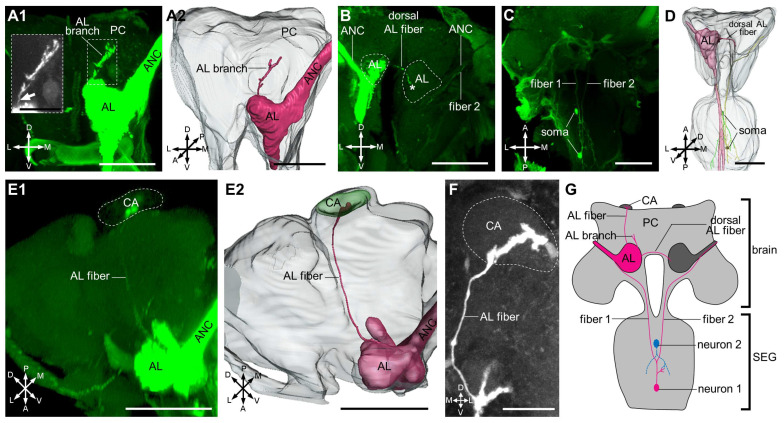
Secondary central projections of the antennal neurons in the brain. (**A1**,**A2**): There is an AL branch which projects to some region of the PC, and the embedded black-and-white image shows the detail of only one nerve fiber (indicates by the arrow). (**B**): There is a dorsal AL fiber which projects through the ipsilateral AL to the contralateral AL (asterisk indicates the terminal) and one fiber (fiber 2) from the ipsilateral ANC to the contralateral ANC. (**C**): Two somas and two fibers (fiber 1 and fiber 2) are projected from ipsilateral antenna. (**D**): Three-dimensional reconstruction showing the projection details in (**B**,**C**). (**E1**,**E2**): There is one fiber (AL fiber) which projects through/from the AL to the ipsilateral CA. Although the weak staining intensity results in poor clarity of the nerve fiber, another image clearly showing a single fiber projecting to the CA is presented in (**F**). (**G**): Sketch of the secondary central projections of antennal neurons in the brain and SEG. Neuron 1 bifurcates into two fibers projecting toward the antennae and is putatively involved in the rapid processing of antennal olfactory signals and mouthpart gustatory signals. Neuron 2 (dashed outline) cannot be confirmed to be entirely within the SEG. PC, protocerebrum; CA, calyx; AL, antennal lobe; ANC, antennal nerve cord; SEG, subesophageal ganglion; A, anterior; D, dorsal; L, lateral; M, medial; P, posterior; V, ventral. Scale bars = 50 μm, except the black-and-white images with scale bars = 20 μm.

We identified several neural fibers, including a branch from the AL to some uncertain parts of the protocerebrum (Figure 5A1,A2), a neural fiber projecting from the ipsilateral AL to the contralateral AL (Figure 5B,D), and one neural fiber projecting through/from the AL to the ipsilateral calyx (Figure 5E1,E2,F). Moreover, we identified two somas within the ganglia. One neuron appeared to be located within the subesophageal ganglion, whereas the other projected bilaterally to the antennal nerves (Figure 5C,D,G).

## 4. Discussion

In this study, we successfully reconstructed a three-dimensional structure of the central nervous system in *M. persicae*, providing a foundational framework for visualizing the pathways of the antennal nerve. Our investigation elucidated intricated central projection patterns of the antennal nerve within the whole central nervous system.

Our data demonstrated that antennal neuron fibers exhibited a consistent pattern by projecting via the antennal nerve to the ipsilateral side of the central nervous system, including the AL, protocerebrum, AMMC, and subesophageal ganglion, as well as the prothoracic, mesothoracic, and metathoracic neuromeres. A similar projection pattern has been observed in the aphid *Acyrthosiphon pisum*, the blood-sucking bug *Rhodnius prolixus* and the mirid bug *Apolygus lucorum* [33,35,36]. Upon examination within the AL, we observed a notable absence of distinct glomeruli based on synapsin immunostaining, a pattern consistent with findings for *A. pisum* from Gadenne et al. [34]. However, neuronal tracer staining revealed that the antennal sensory neuron axon terminals exhibited well-defined glomerular outlines, aligning with results for *A. pisum* from Kollmann et al. [33]. The recognition and characterization of glomeruli within the AL of aphids suggested a more systematic and thorough investigation to better understand this intriguing aspect of their sensory processing.

Our reconstruction unveiled the presence of a neural fiber projecting to the contralateral AL, suggesting potential bilateral projection patterns similar to that observed in *Drosophila* [17]. This bilateral projection may underlie the functional basis for rapid, odor-directed turns, akin to observations in flies [16]. However, the precise nature of this projection remains uncertain—whether the neural fiber traverses through the contralateral AL or terminates within it. Confirmation of this structural detail may require advanced techniques like serial section transmission electron microscopy for a more in-depth and accurate understanding.

Another notable finding was a thin neural fiber projecting through/from the AL to the calyx, potentially serving a distinct function. The slender nature of this fiber could explain why researchers encountered challenges in observing its terminus [33]. Given the association of calyx with the insect odor memory [37,38,39], the potential involvement of this neural connection in memory processes adds an intriguing layer to our understanding of sensory and cognitive functions within the aphid’s central nervous system. Additionally, a neural fiber branch from AL extended to unidentified regions within the protocerebrum, promoting further investigation into this area and its function.

Antennal nerve fibers posterior to the AL projected to the AMMC, which is responsible for antenna movement control [25,33,35,36,40,41,42,43,44,45]. Subsequently, a subset of fibers descended to the subesophageal ganglion, known as the gustatory center [46,47]. In our data, we observed that one neuron appeared to be confined within the subesophageal ganglion (Figure 5C,D,G). This may be attributed to electrical synapses (gap junctions) between this neuron and other antennal nerve fibers, as the Micro-Ruby neural tracer is membrane impermeable. Alternatively, this neuron was not fully visualized, since mass staining makes it difficult to completely trace the entire nerve fibers. Finally, the antennal nerve projects to the motor area of the thoracic ganglia [48], implying a close connection with motor neurons. This observation suggests a potential role in coordinating motor responses based on sensory input from the antennae.

Taken together, our findings provide a comprehensive map of central projections originating from the antenna in aphid *M. persicae*. This map holds the potential to enhance our understanding of how sensory information is processed and integrated in the central nervous system. To delve deeper into the intricate structures of aphid central nervous system neurons, we propose employing the technique of serial section transmission electron microscopy. This advanced method has the capability to reveal the actual structures, laying a solid foundation for subsequent investigations into the intricacies of aphid neural circuitry.

## Data Availability

The data that support the findings of this study are available from the corresponding authors upon request.
